# (*OC*-6-33)-(2,2′-Bipyridine-κ^2^ *N*,*N*′)trimeth­yl(2-methyl­sulfanyl-2-thia­zoline-κ*N*)platinum(IV) tetra­fluoridoborate

**DOI:** 10.1107/S1600536810027546

**Published:** 2010-07-17

**Authors:** Cornelia Vetter, Clemens Bruhn, Dirk Steinborn

**Affiliations:** a Institut für Chemie – Anorganische Chemie, Martin-Luther-Universität, Halle-Wittenberg, D-06120 Halle, Kurt-Mothes-Strasse 2, Germany; b Institut für Chemie, Universität Kassel, D-34132 Kassel, Heinrich-Plett-Strasse 40, Germany

## Abstract

The asymmetric unit of the title complex, [Pt(CH_3_)_3_(C_10_H_8_N_2_)(C_4_H_7_NS_2_)]BF_4_, contains two crystallographically independent mol­ecules. The Pt^IV^ atom in each complex cation exhibits a distorted octa­hedral coordination geometry, built up by three methyl ligands in a facial binding fashion, a bipyridine ligand and a monodentately *N*-bound 2-methyl­sulfanyl-2-thia­zoline ligand (configuration index: *OC*-6–33). In the crystal structure, weak inter­molecular C—H⋯F hydrogen bonds are found between the complex cations and BF_4_^−^ anions.

## Related literature

For general background to the substitution reactions starting from complexes exhibiting a PtMe_3_ unit, see: Clegg *et al.* (1972 ▶); Lindner *et al.* (2008 ▶); Steinborn & Junicke (2000 ▶); Vetter *et al.* (2006 ▶, 2010 ▶). For a description of the Cambridge Structural Database, see: Allen (2002 ▶). For the conformation of the five-membered thia­zoline ring, see: Bucourt (1974 ▶). For the ligand synthesis, see: Bose *et al.* (1973 ▶).
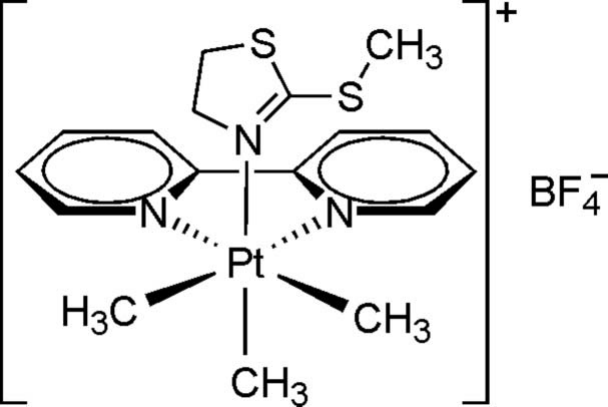


## Experimental

### 

#### Crystal data

[Pt(CH_3_)_3_(C_10_H_8_N_2_)(C_4_H_7_NS_2_)]BF_4_
*M* *_r_* = 616.41
Triclinic, 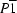

*a* = 10.5163 (8) Å
*b* = 13.2441 (11) Å
*c* = 17.1372 (14) Å
α = 106.776 (6)°
β = 106.690 (6)°
γ = 97.050 (6)°
*V* = 2133.3 (3) Å^3^
*Z* = 4
Mo *K*α radiation
μ = 6.82 mm^−1^
*T* = 173 K
0.55 × 0.30 × 0.26 mm

#### Data collection

Stoe IPDS-2 diffractometer
Absorption correction: numerical (*X-RED*; Stoe & Cie, 2002 ▶) *T*_min_ = 0.082, *T*_max_ = 0.259
15501 measured reflections
7133 independent reflections
6275 reflections with *I* > 2σ(*I*)
*R*_int_ = 0.072

#### Refinement

*R*[*F* ^2^ > 2σ(*F* ^2^)] = 0.045
*wR*(*F* ^2^) = 0.118
*S* = 1.01
7133 reflections
513 parameters
H-atom parameters constrained
Δρ_max_ = 2.72 e Å^−3^
Δρ_min_ = −4.03 e Å^−3^

### 

Data collection: *X-AREA* (Stoe & Cie, 2002 ▶); cell refinement: *X-AREA*; data reduction: *X-RED* (Stoe & Cie, 2002 ▶); program(s) used to solve structure: *SHELXS97* (Sheldrick, 2008 ▶); program(s) used to refine structure: *SHELXL97* (Sheldrick, 2008 ▶); molecular graphics: *DIAMOND* (Brandenburg, 1999 ▶); software used to prepare material for publication: *SHELXL97*.

## Supplementary Material

Crystal structure: contains datablocks I, global. DOI: 10.1107/S1600536810027546/hy2325sup1.cif

Structure factors: contains datablocks I. DOI: 10.1107/S1600536810027546/hy2325Isup2.hkl

Additional supplementary materials:  crystallographic information; 3D view; checkCIF report

## Figures and Tables

**Table 1:** Selected bond lengths (Å)

Pt1—C1	2.062 (8)
Pt1—C2	2.060 (7)
Pt1—C3	2.060 (6)
Pt1—N1	2.222 (5)
Pt1—N2	2.166 (6)
Pt1—N3	2.176 (5)
Pt2—C18	2.061 (7)
Pt2—C19	2.048 (8)
Pt2—C20	2.055 (9)
Pt2—N4	2.245 (6)
Pt2—N5	2.146 (6)
Pt2—N6	2.174 (5)

**Table 2:** Hydrogen-bond geometry (Å, °)

*D*—H⋯*A*	*D*—H	H⋯*A*	*D*⋯*A*	*D*—H⋯*A*
C7—H7*C*⋯F8	0.98	2.45	3.383 (11)	158
C9—H9*A*⋯F1^i^	0.95	2.44	3.192 (10)	136
C16—H16*A*⋯F6^ii^	0.95	2.44	3.114 (9)	128
C17—H17*A*⋯F7^ii^	0.95	2.39	3.190 (9)	142
C28—H28*A*⋯F2^iii^	0.95	2.53	3.322 (10)	141
C32—H32*A*⋯F8^iv^	0.95	2.55	3.497 (11)	173
C34—H34*A*⋯F4	0.95	2.43	3.178 (8)	136

Symmetry codes: (i) 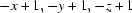; (ii) 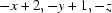; (iii) 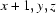; (iv) 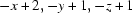.

**Fig. 1.**
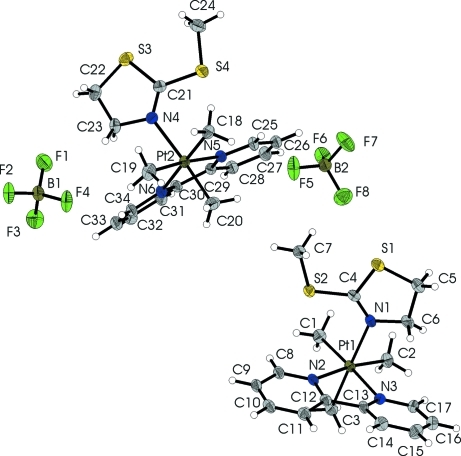
Molecular structure of the title compound. Displacement ellipsoids are drawn at the 50% probability level.

## supplementary crystallographic information

### Comment

Due to the low-spin *d*^6^ electron configuration of platinum(IV), ligand substitution reactions of Pt(IV) complexes may be hampered. Starting from complexes exhibiting a PtMe_3_ unit (Clegg *et al.*, 1972; Lindner *et al.*, 2008; Vetter *et al.*, 2006, 2010), substitution reactions were found to proceed smoothly even with weak donors (Steinborn & Junicke, 2000) because the leaving ligand is additionally activated by the high *trans* effect of a methyl ligand in *trans* position.

The asymmetric unit of the title compound consists of two symmetrically independent, structurally very similar molecules of two cationic Pt(IV) complexes [PtMe_3_(mttz-κ*N*)(bpy)]^+^ (mttz = 2-methylsulfanyl-2-thiazoline, bpy = 2,2'-bipyridine) as well as two BF_4_^-^ anions (Fig. 1). The primary coordination geometry of the Pt^IV^ atom in the cationic complex is built up by three methyl ligands in a *facial* binding fashion, a bpy ligand and a monodentately bound mttz ligand (Table 1). As expected for Pt(IV) complexes, an octahedral coordination geometry was found, which is distorted due to the restricted bite of the bpy ligand [N2—Pt1—N3 = 76.2 (2) and N5—Pt2—N6 = 76.4 (2)°]. The other angles between *cis* arranged ligands are between 84.0 (4) and 101.1 (3)°. Due to the high *trans* influence of the methyl ligands, the Pt1—N1 and Pt2—N4 bonds were found to be considerably longer [2.222 (5) and 2.245 (6) Å] compared to those of other Pt^IV^—N(CH_2_)═C complexes [median: 2.137, lower/upper quartile: 2.048/2.163 Å, 38 observations taken in consideration from CSD (version 5.30, Allen, 2002)]. The conformation of the five-membered thiazoline rings could be described as distorted half chair along C5—C6 and C22—C23, respectively (Bucourt, 1974). In the crystal of the title complex, weak intermolecular C—H···F hydrogen bonds were found between the cationic Pt(IV) complexes and BF_4_^-^ anions (Table 2).

### Experimental

Under anaerobic conditions [PtMe_3_I(bpy)] (70 mg, 0.13 mmol) and AgBF_4_ (26 mg, 0.13 mmol) were stirred in acetone (10 ml) for 30 min under absence of light. The precipitated AgI was filtered off and the colorless, clear filtrate was added to 2-methylsulfanyl-2-thiazoline (18 mg, 0.13 mmol) (Bose *et al.*, 1973). The reaction mixture was stirred for 15 h, then the solvent was reduced in *vacuo* to 1 ml, layered with diethyl ether (3 ml) and cooled to -40°C. After 12 h the title complex was obtained as needles.

### Refinement

All H atoms were positioned geometrically and allowed to ride on the respective parent atoms, with C—H = 0.95–0.99 Å and *U*_iso_(H) = 1.2*U*_eq_(C). The highest residual electron density was found 0.98 Å from Pt2 and the deepest hole 0.89 Å from Pt1.

### Crystal data

**Table:**

[Pt(CH_3_)_3_(C_10_H_8_N_2_)(C_4_H_7_NS_2_)]BF_4_	*Z* = 4
*M**_r_* = 616.41	*F*(000) = 1192
Triclinic, *P*1	*D*_x_ = 1.919 Mg m^−^^3^
Hall symbol: -P 1	Mo *K*α radiation, λ = 0.71073 Å
*a* = 10.5163 (8) Å	Cell parameters from 24955 reflections
*b* = 13.2441 (11) Å	θ = 1.7–25.6°
*c* = 17.1372 (14) Å	µ = 6.82 mm^−^^1^
α = 106.776 (6)°	*T* = 173 K
β = 106.690 (6)°	Block, yellow
γ = 97.050 (6)°	0.55 × 0.30 × 0.26 mm
*V* = 2133.3 (3) Å^3^	

### Data collection

**Table:**

Stoe IPDS-2 diffractometer	7133 independent reflections
Radiation source: sealed X-ray tube, 12 x 0.4 mm long-fine focus	6275 reflections with *I* > 2σ(*I*)
plane graphite	*R*_int_ = 0.072
Detector resolution: 6.67 pixels mm^-1^	θ_max_ = 25.0°, θ_min_ = 1.7°
rotation method scans	*h* = −12→12
Absorption correction: numerical (*X-RED*; Stoe & Cie, 2002)	*k* = −15→15
*T*_min_ = 0.082, *T*_max_ = 0.259	*l* = −20→20
15501 measured reflections	

### Refinement

**Table:**

Refinement on *F*^2^	Primary atom site location: structure-invariant direct methods
Least-squares matrix: full	Secondary atom site location: difference Fourier map
*R*[*F*^2^ > 2σ(*F*^2^)] = 0.045	Hydrogen site location: inferred from neighbouring sites
*wR*(*F*^2^) = 0.118	H-atom parameters constrained
*S* = 1.01	*w* = 1/[σ^2^(*F*_o_^2^) + (0.0874*P*)^2^] where *P* = (*F*_o_^2^ + 2*F*_c_^2^)/3
7133 reflections	(Δ/σ)_max_ = 0.002
513 parameters	Δρ_max_ = 2.72 e Å^−^^3^
0 restraints	Δρ_min_ = −4.03 e Å^−^^3^

### Fractional atomic coordinates and isotropic or equivalent isotropic displacement parameters (Å^2^)

**Table:**

	*x*	*y*	*z*	*U*_iso_*/*U*_eq_	
Pt1	0.90066 (2)	0.795521 (18)	0.128788 (14)	0.03034 (11)	
S1	0.7153 (2)	0.42771 (14)	−0.05713 (12)	0.0476 (5)	
S2	0.6969 (2)	0.54876 (14)	0.11679 (12)	0.0439 (4)	
N1	0.8333 (6)	0.6290 (4)	0.0323 (3)	0.0316 (12)	
N2	0.6950 (6)	0.8095 (4)	0.1220 (4)	0.0325 (12)	
N3	0.8303 (5)	0.8527 (4)	0.0203 (3)	0.0298 (11)	
C1	0.9596 (8)	0.7432 (6)	0.2332 (5)	0.0430 (17)	
H1A	1.0320	0.7996	0.2812	0.065*	
H1B	0.9933	0.6773	0.2166	0.065*	
H1C	0.8814	0.7279	0.2515	0.065*	
C2	1.0985 (8)	0.8014 (7)	0.1312 (5)	0.0441 (18)	
H2A	1.0987	0.7524	0.0761	0.066*	
H2B	1.1487	0.7792	0.1788	0.066*	
H2C	1.1421	0.8754	0.1399	0.066*	
C3	0.9625 (7)	0.9513 (5)	0.2160 (5)	0.0399 (16)	
H3A	0.9384	0.9531	0.2674	0.060*	
H3B	0.9173	0.9993	0.1891	0.060*	
H3C	1.0615	0.9756	0.2329	0.060*	
C4	0.7580 (7)	0.5470 (5)	0.0323 (4)	0.0349 (14)	
C5	0.8328 (8)	0.4902 (6)	−0.0979 (5)	0.0479 (18)	
H5A	0.9196	0.4662	−0.0841	0.057*	
H5B	0.7932	0.4715	−0.1615	0.057*	
C6	0.8557 (9)	0.6106 (6)	−0.0524 (4)	0.049 (2)	
H6A	0.9498	0.6469	−0.0427	0.058*	
H6B	0.7917	0.6413	−0.0888	0.058*	
C7	0.5935 (8)	0.4148 (6)	0.0833 (5)	0.0464 (18)	
H7A	0.5181	0.4015	0.0296	0.070*	
H7B	0.5570	0.4084	0.1286	0.070*	
H7C	0.6492	0.3614	0.0733	0.070*	
C8	0.6363 (7)	0.7983 (5)	0.1800 (4)	0.0385 (16)	
H8A	0.6831	0.7741	0.2250	0.046*	
C9	0.5110 (7)	0.8206 (5)	0.1766 (5)	0.0390 (16)	
H9A	0.4724	0.8123	0.2188	0.047*	
C10	0.4419 (7)	0.8549 (6)	0.1115 (5)	0.0440 (18)	
H10A	0.3546	0.8697	0.1076	0.053*	
C11	0.5008 (7)	0.8677 (6)	0.0519 (5)	0.0439 (17)	
H11A	0.4555	0.8925	0.0069	0.053*	
C12	0.6281 (7)	0.8436 (5)	0.0584 (4)	0.0337 (14)	
C13	0.7013 (7)	0.8560 (5)	−0.0021 (4)	0.0337 (15)	
C14	0.6371 (8)	0.8755 (6)	−0.0784 (5)	0.0434 (17)	
H14A	0.5430	0.8760	−0.0955	0.052*	
C15	0.7138 (9)	0.8938 (7)	−0.1279 (5)	0.051 (2)	
H15A	0.6729	0.9085	−0.1794	0.061*	
C16	0.8479 (8)	0.8911 (6)	−0.1033 (5)	0.0454 (17)	
H16A	0.9018	0.9037	−0.1370	0.054*	
C17	0.9039 (7)	0.8695 (5)	−0.0282 (4)	0.0367 (15)	
H17A	0.9973	0.8666	−0.0107	0.044*	
Pt2	0.69060 (2)	0.254880 (18)	0.416978 (15)	0.03203 (11)	
S3	0.7284 (2)	0.00166 (16)	0.57064 (12)	0.0508 (5)	
S4	0.8042 (3)	0.00206 (16)	0.41291 (13)	0.0553 (5)	
N4	0.7031 (6)	0.1511 (5)	0.5008 (4)	0.0385 (13)	
N5	0.9006 (6)	0.2658 (4)	0.4277 (4)	0.0336 (13)	
N6	0.7990 (6)	0.3874 (4)	0.5379 (4)	0.0376 (14)	
C18	0.6002 (8)	0.1255 (6)	0.3032 (5)	0.0439 (18)	
H18A	0.6703	0.0907	0.2869	0.066*	
H18B	0.5520	0.1510	0.2572	0.066*	
H18C	0.5354	0.0731	0.3114	0.066*	
C19	0.4940 (8)	0.2598 (8)	0.4102 (6)	0.055 (2)	
H19A	0.4565	0.2017	0.4275	0.083*	
H19B	0.4390	0.2503	0.3507	0.083*	
H19C	0.4923	0.3299	0.4492	0.083*	
C20	0.6776 (10)	0.3554 (7)	0.3447 (7)	0.061 (2)	
H20A	0.5874	0.3335	0.2998	0.091*	
H20B	0.7478	0.3504	0.3173	0.091*	
H20C	0.6916	0.4301	0.3824	0.091*	
C21	0.7396 (7)	0.0620 (5)	0.4931 (4)	0.0384 (15)	
C22	0.6366 (8)	0.1002 (6)	0.6105 (5)	0.0486 (19)	
H22A	0.5375	0.0683	0.5873	0.058*	
H22B	0.6669	0.1252	0.6746	0.058*	
C23	0.6678 (8)	0.1919 (6)	0.5802 (5)	0.0445 (17)	
H23A	0.5876	0.2244	0.5676	0.053*	
H23B	0.7450	0.2484	0.6259	0.053*	
C24	0.8292 (12)	−0.1231 (7)	0.4296 (6)	0.067 (3)	
H24A	0.7406	−0.1726	0.4112	0.101*	
H24B	0.8772	−0.1088	0.4912	0.101*	
H24C	0.8835	−0.1559	0.3956	0.101*	
C25	0.9448 (7)	0.2162 (5)	0.3634 (4)	0.0380 (15)	
H25A	0.8800	0.1706	0.3092	0.046*	
C26	1.0807 (8)	0.2298 (6)	0.3738 (5)	0.0420 (16)	
H26A	1.1095	0.1942	0.3274	0.050*	
C27	1.1754 (8)	0.2958 (6)	0.4526 (5)	0.0454 (17)	
H27A	1.2701	0.3046	0.4617	0.054*	
C28	1.1301 (7)	0.3490 (6)	0.5182 (5)	0.0421 (16)	
H28A	1.1935	0.3957	0.5724	0.051*	
C29	0.9931 (7)	0.3338 (5)	0.5040 (4)	0.0345 (14)	
C30	0.9366 (7)	0.3958 (5)	0.5676 (5)	0.0385 (16)	
C31	1.0144 (8)	0.4619 (6)	0.6503 (5)	0.049 (2)	
H31A	1.1093	0.4646	0.6710	0.059*	
C32	0.9557 (10)	0.5250 (6)	0.7040 (5)	0.061 (2)	
H32A	1.0088	0.5708	0.7613	0.073*	
C33	0.8183 (10)	0.5189 (6)	0.6712 (5)	0.056 (2)	
H33A	0.7755	0.5627	0.7055	0.068*	
C34	0.7431 (9)	0.4500 (6)	0.5896 (5)	0.048 (2)	
H34A	0.6480	0.4462	0.5685	0.058*	
F1	0.5476 (9)	0.3215 (6)	0.7103 (5)	0.102 (2)	
F2	0.3580 (5)	0.3890 (5)	0.7105 (3)	0.0725 (16)	
F3	0.5624 (6)	0.4994 (5)	0.7516 (3)	0.0755 (16)	
F4	0.4545 (5)	0.3968 (5)	0.6108 (3)	0.0651 (15)	
B1	0.4768 (10)	0.3997 (8)	0.6943 (6)	0.048 (2)	
F5	0.7192 (6)	0.2028 (6)	0.1475 (4)	0.089 (2)	
F6	0.9447 (5)	0.2042 (5)	0.1846 (3)	0.0673 (14)	
F7	0.8041 (6)	0.1086 (5)	0.0505 (4)	0.084 (2)	
F8	0.8528 (7)	0.2856 (5)	0.0919 (4)	0.0819 (17)	
B2	0.8268 (10)	0.1985 (7)	0.1203 (6)	0.045 (2)	

### Atomic displacement parameters (Å^2^)

**Table:**

	*U*^11^	*U*^22^	*U*^33^	*U*^12^	*U*^13^	*U*^23^
Pt1	0.02865 (17)	0.03093 (15)	0.02646 (15)	0.00606 (11)	0.00803 (11)	0.00410 (11)
S1	0.0597 (12)	0.0337 (8)	0.0392 (9)	0.0042 (8)	0.0200 (9)	−0.0030 (7)
S2	0.0535 (11)	0.0355 (8)	0.0407 (9)	0.0052 (8)	0.0232 (8)	0.0050 (7)
N1	0.028 (3)	0.035 (3)	0.026 (3)	0.009 (2)	0.007 (2)	0.003 (2)
N2	0.032 (3)	0.029 (2)	0.032 (3)	0.005 (2)	0.013 (2)	0.003 (2)
N3	0.027 (3)	0.030 (2)	0.026 (2)	0.004 (2)	0.004 (2)	0.007 (2)
C1	0.045 (4)	0.047 (4)	0.033 (3)	0.011 (3)	0.009 (3)	0.012 (3)
C2	0.030 (4)	0.055 (4)	0.040 (4)	0.006 (3)	0.010 (3)	0.010 (3)
C3	0.035 (4)	0.037 (3)	0.041 (4)	0.009 (3)	0.009 (3)	0.007 (3)
C4	0.033 (3)	0.031 (3)	0.033 (3)	0.010 (3)	0.007 (3)	0.004 (3)
C5	0.049 (4)	0.050 (4)	0.038 (4)	0.013 (4)	0.015 (3)	0.006 (3)
C6	0.078 (6)	0.036 (3)	0.025 (3)	0.012 (4)	0.020 (4)	−0.003 (3)
C7	0.048 (4)	0.038 (4)	0.056 (4)	0.009 (3)	0.024 (4)	0.014 (3)
C8	0.043 (4)	0.036 (3)	0.034 (3)	0.006 (3)	0.019 (3)	0.004 (3)
C9	0.038 (4)	0.039 (3)	0.044 (4)	0.009 (3)	0.023 (3)	0.010 (3)
C10	0.032 (4)	0.042 (4)	0.052 (4)	0.009 (3)	0.018 (3)	0.004 (3)
C11	0.041 (4)	0.040 (4)	0.048 (4)	0.009 (3)	0.017 (3)	0.010 (3)
C12	0.031 (3)	0.030 (3)	0.032 (3)	0.001 (3)	0.009 (3)	0.003 (2)
C13	0.035 (4)	0.030 (3)	0.027 (3)	0.001 (3)	0.012 (3)	−0.002 (2)
C14	0.035 (4)	0.050 (4)	0.043 (4)	0.011 (3)	0.010 (3)	0.015 (3)
C15	0.057 (5)	0.057 (4)	0.036 (4)	0.008 (4)	0.009 (3)	0.019 (3)
C16	0.045 (4)	0.053 (4)	0.042 (4)	0.012 (4)	0.019 (3)	0.017 (3)
C17	0.038 (4)	0.041 (3)	0.032 (3)	0.006 (3)	0.013 (3)	0.014 (3)
Pt2	0.02815 (17)	0.03542 (16)	0.03201 (16)	0.00603 (11)	0.01136 (12)	0.01006 (11)
S3	0.0646 (13)	0.0493 (10)	0.0361 (9)	0.0065 (9)	0.0116 (9)	0.0192 (8)
S4	0.0844 (16)	0.0482 (10)	0.0480 (10)	0.0291 (10)	0.0350 (11)	0.0195 (8)
N4	0.043 (3)	0.038 (3)	0.035 (3)	0.005 (3)	0.016 (3)	0.011 (2)
N5	0.030 (3)	0.033 (3)	0.034 (3)	0.005 (2)	0.012 (2)	0.006 (2)
N6	0.035 (3)	0.034 (3)	0.042 (3)	0.002 (2)	0.018 (3)	0.007 (2)
C18	0.043 (4)	0.050 (4)	0.035 (3)	0.004 (3)	0.009 (3)	0.016 (3)
C19	0.024 (4)	0.075 (6)	0.066 (5)	0.007 (4)	0.014 (4)	0.027 (5)
C20	0.063 (6)	0.051 (5)	0.079 (6)	0.014 (4)	0.031 (5)	0.031 (4)
C21	0.041 (4)	0.037 (3)	0.032 (3)	0.005 (3)	0.006 (3)	0.012 (3)
C22	0.048 (4)	0.060 (5)	0.032 (3)	0.003 (4)	0.013 (3)	0.011 (3)
C23	0.048 (4)	0.055 (4)	0.032 (3)	0.012 (4)	0.019 (3)	0.011 (3)
C24	0.101 (8)	0.048 (4)	0.058 (5)	0.030 (5)	0.031 (5)	0.016 (4)
C25	0.038 (4)	0.043 (3)	0.035 (3)	0.009 (3)	0.019 (3)	0.008 (3)
C26	0.044 (4)	0.048 (4)	0.044 (4)	0.019 (3)	0.022 (3)	0.018 (3)
C27	0.034 (4)	0.054 (4)	0.053 (4)	0.013 (3)	0.020 (3)	0.018 (4)
C28	0.037 (4)	0.042 (4)	0.042 (4)	0.003 (3)	0.014 (3)	0.008 (3)
C29	0.034 (4)	0.034 (3)	0.035 (3)	0.006 (3)	0.013 (3)	0.011 (3)
C30	0.035 (4)	0.031 (3)	0.050 (4)	0.005 (3)	0.022 (3)	0.008 (3)
C31	0.049 (4)	0.042 (4)	0.040 (4)	−0.006 (3)	0.018 (3)	−0.004 (3)
C32	0.076 (6)	0.043 (4)	0.047 (4)	−0.007 (4)	0.026 (4)	−0.006 (4)
C33	0.073 (6)	0.041 (4)	0.056 (5)	0.003 (4)	0.041 (5)	0.002 (4)
C34	0.055 (5)	0.038 (3)	0.050 (4)	0.004 (3)	0.033 (4)	0.000 (3)
F1	0.163 (7)	0.105 (5)	0.092 (5)	0.085 (5)	0.076 (5)	0.056 (4)
F2	0.065 (3)	0.108 (4)	0.048 (3)	0.014 (3)	0.035 (2)	0.019 (3)
F3	0.066 (3)	0.085 (4)	0.057 (3)	0.005 (3)	0.010 (3)	0.013 (3)
F4	0.042 (3)	0.117 (4)	0.039 (2)	0.019 (3)	0.019 (2)	0.026 (3)
B1	0.048 (5)	0.060 (5)	0.043 (4)	0.017 (4)	0.022 (4)	0.018 (4)
F5	0.067 (3)	0.152 (6)	0.063 (3)	0.034 (4)	0.040 (3)	0.036 (4)
F6	0.058 (3)	0.091 (4)	0.049 (3)	0.033 (3)	0.009 (2)	0.022 (3)
F7	0.059 (3)	0.075 (3)	0.078 (4)	0.011 (3)	0.018 (3)	−0.024 (3)
F8	0.080 (4)	0.085 (4)	0.096 (4)	0.027 (3)	0.030 (4)	0.048 (4)
B2	0.048 (5)	0.053 (5)	0.041 (4)	0.019 (4)	0.017 (4)	0.021 (4)

### Geometric parameters (Å, °)

**Table:**

Pt1—C1	2.062 (8)	Pt2—N5	2.146 (6)
Pt1—C2	2.060 (7)	Pt2—N6	2.174 (5)
Pt1—C3	2.060 (6)	S3—C21	1.760 (7)
Pt1—N1	2.222 (5)	S3—C22	1.809 (9)
Pt1—N2	2.166 (6)	S4—C21	1.738 (7)
Pt1—N3	2.176 (5)	S4—C24	1.799 (9)
S1—C4	1.755 (6)	N4—C21	1.269 (10)
S1—C5	1.811 (8)	N4—C23	1.483 (8)
S2—C4	1.740 (6)	N5—C25	1.346 (8)
S2—C7	1.800 (7)	N5—C29	1.358 (9)
N1—C4	1.264 (8)	N6—C34	1.346 (9)
N1—C6	1.493 (8)	N6—C30	1.368 (9)
N2—C12	1.344 (9)	C18—H18A	0.9800
N2—C8	1.345 (8)	C18—H18B	0.9800
N3—C13	1.310 (9)	C18—H18C	0.9800
N3—C17	1.332 (8)	C19—H19A	0.9800
C1—H1A	0.9800	C19—H19B	0.9800
C1—H1B	0.9800	C19—H19C	0.9800
C1—H1C	0.9800	C20—H20A	0.9800
C2—H2A	0.9800	C20—H20B	0.9800
C2—H2B	0.9800	C20—H20C	0.9800
C2—H2C	0.9800	C22—C23	1.489 (11)
C3—H3A	0.9800	C22—H22A	0.9900
C3—H3B	0.9800	C22—H22B	0.9900
C3—H3C	0.9800	C23—H23A	0.9900
C5—C6	1.512 (10)	C23—H23B	0.9900
C5—H5A	0.9900	C24—H24A	0.9800
C5—H5B	0.9900	C24—H24B	0.9800
C6—H6A	0.9900	C24—H24C	0.9800
C6—H6B	0.9900	C25—C26	1.370 (10)
C7—H7A	0.9800	C25—H25A	0.9500
C7—H7B	0.9800	C26—C27	1.383 (11)
C7—H7C	0.9800	C26—H26A	0.9500
C8—C9	1.374 (10)	C27—C28	1.385 (10)
C8—H8A	0.9500	C27—H27A	0.9500
C9—C10	1.374 (11)	C28—C29	1.369 (10)
C9—H9A	0.9500	C28—H28A	0.9500
C10—C11	1.376 (10)	C29—C30	1.480 (9)
C10—H10A	0.9500	C30—C31	1.373 (10)
C11—C12	1.395 (10)	C31—C32	1.391 (11)
C11—H11A	0.9500	C31—H31A	0.9500
C12—C13	1.489 (8)	C32—C33	1.375 (14)
C13—C14	1.400 (10)	C32—H32A	0.9500
C14—C15	1.376 (10)	C33—C34	1.366 (11)
C14—H14A	0.9500	C33—H33A	0.9500
C15—C16	1.360 (12)	C34—H34A	0.9500
C15—H15A	0.9500	F1—B1	1.391 (12)
C16—C17	1.380 (11)	F2—B1	1.356 (10)
C16—H16A	0.9500	F3—B1	1.403 (11)
C17—H17A	0.9500	F4—B1	1.370 (10)
Pt2—C18	2.061 (7)	F5—B2	1.342 (10)
Pt2—C19	2.048 (8)	F6—B2	1.379 (11)
Pt2—C20	2.055 (9)	F7—B2	1.360 (10)
Pt2—N4	2.245 (6)	F8—B2	1.401 (11)
			
C3—Pt1—C2	88.8 (3)	C19—Pt2—N6	99.8 (3)
C3—Pt1—C1	87.6 (3)	C20—Pt2—N6	92.9 (3)
C2—Pt1—C1	85.0 (3)	C18—Pt2—N6	176.0 (3)
C3—Pt1—N2	86.5 (2)	N5—Pt2—N6	76.4 (2)
C2—Pt1—N2	172.1 (3)	C19—Pt2—N4	91.2 (3)
C1—Pt1—N2	101.1 (3)	C20—Pt2—N4	177.3 (3)
C3—Pt1—N3	91.6 (3)	C18—Pt2—N4	93.7 (3)
C2—Pt1—N3	97.6 (3)	N5—Pt2—N4	92.1 (2)
C1—Pt1—N3	177.3 (2)	N6—Pt2—N4	85.0 (2)
N2—Pt1—N3	76.2 (2)	C21—S3—C22	89.1 (4)
C3—Pt1—N1	178.7 (2)	C21—S4—C24	103.3 (4)
C2—Pt1—N1	91.1 (3)	C21—N4—C23	112.4 (6)
C1—Pt1—N1	93.7 (3)	C21—N4—Pt2	130.6 (5)
N2—Pt1—N1	93.46 (19)	C23—N4—Pt2	117.0 (5)
N3—Pt1—N1	87.1 (2)	C25—N5—C29	119.0 (6)
C4—S1—C5	89.9 (3)	C25—N5—Pt2	125.0 (5)
C4—S2—C7	104.4 (3)	C29—N5—Pt2	115.9 (4)
C4—N1—C6	111.8 (5)	C34—N6—C30	118.5 (6)
C4—N1—Pt1	128.6 (4)	C34—N6—Pt2	126.7 (5)
C6—N1—Pt1	118.6 (4)	C30—N6—Pt2	114.2 (4)
C12—N2—C8	118.6 (6)	Pt2—C18—H18A	109.5
C12—N2—Pt1	115.2 (4)	Pt2—C18—H18B	109.5
C8—N2—Pt1	125.8 (5)	H18A—C18—H18B	109.5
C13—N3—C17	120.7 (6)	Pt2—C18—H18C	109.5
C13—N3—Pt1	114.5 (4)	H18A—C18—H18C	109.5
C17—N3—Pt1	124.2 (5)	H18B—C18—H18C	109.5
Pt1—C1—H1A	109.5	Pt2—C19—H19A	109.5
Pt1—C1—H1B	109.5	Pt2—C19—H19B	109.5
H1A—C1—H1B	109.5	H19A—C19—H19B	109.5
Pt1—C1—H1C	109.5	Pt2—C19—H19C	109.5
H1A—C1—H1C	109.5	H19A—C19—H19C	109.5
H1B—C1—H1C	109.5	H19B—C19—H19C	109.5
Pt1—C2—H2A	109.5	Pt2—C20—H20A	109.5
Pt1—C2—H2B	109.5	Pt2—C20—H20B	109.5
H2A—C2—H2B	109.5	H20A—C20—H20B	109.5
Pt1—C2—H2C	109.5	Pt2—C20—H20C	109.5
H2A—C2—H2C	109.5	H20A—C20—H20C	109.5
H2B—C2—H2C	109.5	H20B—C20—H20C	109.5
Pt1—C3—H3A	109.5	N4—C21—S4	122.7 (5)
Pt1—C3—H3B	109.5	N4—C21—S3	117.1 (5)
H3A—C3—H3B	109.5	S4—C21—S3	120.2 (4)
Pt1—C3—H3C	109.5	C23—C22—S3	106.2 (5)
H3A—C3—H3C	109.5	C23—C22—H22A	110.5
H3B—C3—H3C	109.5	S3—C22—H22A	110.5
N1—C4—S2	122.6 (5)	C23—C22—H22B	110.5
N1—C4—S1	117.6 (5)	S3—C22—H22B	110.5
S2—C4—S1	119.8 (4)	H22A—C22—H22B	108.7
C6—C5—S1	105.1 (5)	N4—C23—C22	109.0 (6)
C6—C5—H5A	110.7	N4—C23—H23A	109.9
S1—C5—H5A	110.7	C22—C23—H23A	109.9
C6—C5—H5B	110.7	N4—C23—H23B	109.9
S1—C5—H5B	110.7	C22—C23—H23B	109.9
H5A—C5—H5B	108.8	H23A—C23—H23B	108.3
N1—C6—C5	108.9 (6)	S4—C24—H24A	109.5
N1—C6—H6A	109.9	S4—C24—H24B	109.5
C5—C6—H6A	109.9	H24A—C24—H24B	109.5
N1—C6—H6B	109.9	S4—C24—H24C	109.5
C5—C6—H6B	109.9	H24A—C24—H24C	109.5
H6A—C6—H6B	108.3	H24B—C24—H24C	109.5
S2—C7—H7A	109.5	N5—C25—C26	121.9 (6)
S2—C7—H7B	109.5	N5—C25—H25A	119.1
H7A—C7—H7B	109.5	C26—C25—H25A	119.1
S2—C7—H7C	109.5	C25—C26—C27	119.2 (6)
H7A—C7—H7C	109.5	C25—C26—H26A	120.4
H7B—C7—H7C	109.5	C27—C26—H26A	120.4
N2—C8—C9	122.3 (7)	C26—C27—C28	119.0 (7)
N2—C8—H8A	118.8	C26—C27—H27A	120.5
C9—C8—H8A	118.8	C28—C27—H27A	120.5
C8—C9—C10	119.3 (6)	C29—C28—C27	119.4 (6)
C8—C9—H9A	120.3	C29—C28—H28A	120.3
C10—C9—H9A	120.3	C27—C28—H28A	120.3
C9—C10—C11	119.2 (7)	N5—C29—C28	121.4 (6)
C9—C10—H10A	120.4	N5—C29—C30	116.0 (6)
C11—C10—H10A	120.4	C28—C29—C30	122.4 (6)
C10—C11—C12	119.0 (8)	C31—C30—N6	120.5 (6)
C10—C11—H11A	120.5	C31—C30—C29	123.9 (7)
C12—C11—H11A	120.5	N6—C30—C29	115.6 (6)
N2—C12—C11	121.5 (6)	C30—C31—C32	120.6 (8)
N2—C12—C13	115.2 (6)	C30—C31—H31A	119.7
C11—C12—C13	123.2 (7)	C32—C31—H31A	119.7
N3—C13—C14	120.7 (6)	C33—C32—C31	117.7 (7)
N3—C13—C12	117.6 (6)	C33—C32—H32A	121.1
C14—C13—C12	121.7 (6)	C31—C32—H32A	121.1
C15—C14—C13	118.4 (7)	C34—C33—C32	120.2 (7)
C15—C14—H14A	120.8	C34—C33—H33A	119.9
C13—C14—H14A	120.8	C32—C33—H33A	119.9
C16—C15—C14	120.1 (7)	N6—C34—C33	122.3 (8)
C16—C15—H15A	119.9	N6—C34—H34A	118.8
C14—C15—H15A	119.9	C33—C34—H34A	118.8
C15—C16—C17	118.4 (7)	F2—B1—F4	110.8 (8)
C15—C16—H16A	120.8	F2—B1—F1	112.5 (8)
C17—C16—H16A	120.8	F4—B1—F1	110.1 (7)
N3—C17—C16	121.6 (7)	F2—B1—F3	108.5 (7)
N3—C17—H17A	119.2	F4—B1—F3	109.2 (7)
C16—C17—H17A	119.2	F1—B1—F3	105.6 (8)
C19—Pt2—C20	87.6 (4)	F5—B2—F7	112.4 (8)
C19—Pt2—C18	84.0 (4)	F5—B2—F6	112.9 (7)
C20—Pt2—C18	88.5 (3)	F7—B2—F6	109.1 (8)
C19—Pt2—N5	174.7 (3)	F5—B2—F8	110.3 (8)
C20—Pt2—N5	89.0 (3)	F7—B2—F8	104.9 (7)
C18—Pt2—N5	99.9 (3)	F6—B2—F8	106.7 (7)

### Hydrogen-bond geometry (Å, °)

**Table:**

*D*—H···*A*	*D*—H	H···*A*	*D*···*A*	*D*—H···*A*
C7—H7C···F8	0.98	2.45	3.383 (11)	158
C9—H9A···F1^i^	0.95	2.44	3.192 (10)	136
C16—H16A···F6^ii^	0.95	2.44	3.114 (9)	128
C17—H17A···F7^ii^	0.95	2.39	3.190 (9)	142
C28—H28A···F2^iii^	0.95	2.53	3.322 (10)	141
C32—H32A···F8^iv^	0.95	2.55	3.497 (11)	173
C34—H34A···F4	0.95	2.43	3.178 (8)	136

Symmetry codes: (i) −*x*+1, −*y*+1, −*z*+1; (ii) −*x*+2, −*y*+1, −*z*; (iii) *x*+1, *y*, *z*; (iv) −*x*+2, −*y*+1, −*z*+1.

## References

[bb1] Allen, F. H. (2002). *Acta Cryst.* B**58**, 380–388. 10.1107/s0108768102003890 12037359

[bb2] Bose, A. K., Fahey, J. L. & Manhas, M. S. (1973). *J. Heterocycl. Chem.* **10**, 791–794.

[bb3] Brandenburg, K. (1999). *DIAMOND.* Crystal Impact GbR, Bonn, Germany.

[bb4] Bucourt, R. (1974). *Top. Stereochem.* **8**, 159–224.

[bb5] Clegg, D. E., Hall, J. R. & Swile, G. A. (1972). *J. Organomet. Chem.* **38**, 403–420.

[bb6] Lindner, R., Kaludđerović, G. N., Paschke, R., Wagner, C. & Steinborn, D. (2008). *Polyhedron*, **27**, 914–922.

[bb7] Sheldrick, G. M. (2008). *Acta Cryst.* A**64**, 112–122. 10.1107/S0108767307043930 18156677

[bb8] Steinborn, D. & Junicke, H. (2000). *Chem. Rev.* **100**, 4283–4318. 10.1021/cr9903050 11749349

[bb9] Stoe & Cie (2002). *X-AREA* and *X-RED* Stoe & Cie, Darmstadt, Germany.

[bb10] Vetter, C., Wagner, C., Schmidt, J. & Steinborn, D. (2006). *Inorg. Chim. Acta*, **359**, 4326–4334.

[bb11] Vetter, C., Wagner, C. & Steinborn, D. (2010). *Acta Cryst.* E**66**, m286. 10.1107/S160053681000499X PMC2983493 21580231

